# Ultrasound use in metastatic breast cancer to measure body composition changes following an exercise intervention

**DOI:** 10.1038/s41598-021-88375-5

**Published:** 2021-04-23

**Authors:** Adrian Escriche-Escuder, Manuel Trinidad-Fernández, Bella Pajares, Marcos Iglesias-Campos, Emilio Alba, Antonio I. Cuesta-Vargas, Cristina Roldán-Jiménez

**Affiliations:** 1 Department of Physiotherapy, University of Malaga, C/Arquitecto Peñalosa, 3, 29071 Málaga, Spain; 2 Instituto de Investigación Biomédica de Málaga (IBIMA), Málaga, Spain; 3 UGCI Oncológica Médica, Hospital Regional Universitario y Virgen de la Victoria, Málaga, Spain; 4 School of Clinical Sciences, Faculty of Health, Queensland University of Technology, Brisbane, QLD Australia

**Keywords:** Cancer, Biomarkers, Oncology

## Abstract

Changes in body composition and muscle dysfunction are common in metastatic breast cancer (MBC). Ultrasound imaging (US) offers reliable information about muscle and fat tissue architecture (thickness) and quality (echo-intensity). This study aimed to analyze the responsiveness of thickness and echo-intensity and its possible relationship with functional and patient reported-outcomes (PRO) in MBC patients after an exercise intervention. A prospective study was conducted in 2019. A 12-week exercise program was performed, including aerobic exercise and strength training. Measurements were made at baseline and after intervention. Thickness and echo-intensity were obtained from the quadriceps and biceps brachii and brachialis (BB). Mean differences were calculated using the T-Student parametric test for dependent samples of the differences in the means before and after the intervention (p = 0.05; 95% CI). Data from 13 MBC patients showed that some US muscle variables had significant differences after intervention. Best correlations were found between the quality of life questionnaire (QLQ-BR23) PRO and variables from BB muscle thickness in contraction (r = 0.61, p < 0.01), and Non-contraction (r = 0.55, p < 0.01). BB Muscle Non-contraction Thickness also explained 70% of QLQ-BR23 variance. In conclusion, muscle architecture biomarkers showed great responsiveness and are correlated with PRO after an exercise intervention in MBC patients.

## Introduction

Breast cancer (BC) is the most incident cancer for women. With an estimated 1.7 million (95% UI, 1.6–1.78 million) incident cases, in 2016 BC was the leading cause of cancer deaths and disability-adjusted life-years (DALYs) (535,000 deaths and 14.9 million DALYs)^1^. The increased survival to BC achieved due to the medical advances for the last 30 years (approximately 40%)^2^ collides with an increased incidence of metastatic disease. At the time of diagnosis, 3–4% of patients already show metastatic breast cancer (MBC)^3,4^, while up to 30% of women who are diagnosed at an early stage of the disease end up developing metastases after some months or years^5^. Although medical advances have lengthened the survival of patients with MBC^6^, this disease is still considered incurable and is responsible for a significant part of cancer-related deaths^7,8^.

Both in the BC survivors and those patients diagnosed with MBC, most of them remain with signs, symptoms, and poor values of functional upper^9^ and lower limbs capacity and quality of life^10^, as well as significant levels of pain, fatigue, lymphedema or peripheral nervous system disorders^11^. These effects have been related to some treatments used^12^, but may also be aggravated by physical inactivity as observed in other populations^13^. Following the same line of thinking, changes in body composition and muscle dysfunction are also frequent in cancer. Sarcopenia and increased fat tissue have been related to low functional capacity and muscular strength^14^, increased risk of death^15^, and a higher prevalence of treatment side effects^16^. In addition, these consequences are more striking in patients with metastases^17^.

Systematic reviews and meta-analyzes have supported the benefits of exercise, and especially strength training, on quality of life (QoL)^18^, muscle function and aerobic capacity^19^, and cancer-related fatigue in patients with cancer^18,20^. Furthermore, exercise interventions can also improve the parameters of architecture and muscle composition^21,22^. These interventions have been shown to be effective both in the post-treatment and in patients undergoing treatment^23^. Nonetheless, due to the characteristics of the metastatic disease itself, many treatment efforts focus on slowing down the physical and functional deterioration, often inevitable as the disease progresses^24^.

Imaging techniques are considered a useful option for the assessment of sarcopenia. Thus, some affordable and non-invasive tools such as ultrasound imaging (US) can offer reliable and valuable information about tissue architecture (thickness) and quality echo-intensity^25–27^, and these outcomes have been related to strength and functional capacity in some populations^28,29^.

Even though technological development and associated research have suggested US as a potential analysis tool for the body tissue assessment in sarcopenia and other situations, a more significant number of studies are still needed to validate its use in different clinical populations^30^. In patients with MBC, there is a lack of studies assessing muscular architecture and quality with this approach.

### Objective

The purpose of this study was to analyze for the first time the responsiveness of new biomarkers extracted from the US images after an exercise intervention and to study its possible relationship with functional and patient reported-outcomes (PRO) in MBC patients.

## Methods

### Study design and setting

An exercise intervention study was conducted during 2019 at the University Clinical Hospital Virgen de la Victoria in Málaga (Spain).

This study was approved by the Portal de Ética de la Investigación Biomédica de Andalucía Ethics Committee (2804/2016) and registered in ClinicalTrials.gov (NCT03879096) prior to enrollment of participants. The study followed the CONSORT checklist to ensure transparent and standardized reporting of the work. All participants were informed about the purpose and content of the investigation and signed written informed consent form before the start of the study procedures. They were allowed to leave the study when they wanted. The study adhered to the principles of the Declaration of Helsinki. The person appearing in the images have expressly given their informed consent for its publication in an online open access publication.

### Participants

Medical oncologists from the Medical Oncology Unit at University Clinical Hospital Virgen de la Victoria (Malaga, Spain) recruited potentially eligible patients with MBC.

#### Selection criteria

The inclusion criteria were: (1) women older than 18 years, (2) current diagnosis of MBC, not amenable to curative treatment.

Participants were excluded if they had: (1) Previous history of a cardiovascular event during the previous year, including stable or unstable angina; acute pulmonary edema; cardiac rhythm disorders; or syncope of an unknown cause.

### Intervention

A 12-week therapeutic exercise program was performed, including aerobic exercise and strength training led by a physical therapist. Twice a week, the program included 30 min of strength exercises and 20 min of endurance training. The program was executed in group sessions of 7–8 participants. To ensure proper individualization of the program, the evaluators assess the functional capacity individually, adapting the level and exercises performed by each participant to their abilities. Intensity, time, and time prescriptions were individualized based on evaluations of muscular strength, endurance, and patient's needs^31^. In the case of bone metastasis, adaptations were made to avoid specific loading sites^32^. Also, current recommendations in the field of oncology exercise were taken into account^20^.

### Measurements

There were two measurements in the study: at baseline (before intervention) and after a 12-week intervention. An initial assessment session was held in which demographic, medical, and oncological variables were evaluated, as well as functional and image tests. The resultant outcomes were used to extract outcome variables and assess the participant's functional capacity to adjust the exercises and intensity of the therapeutic exercise program. After 12 weeks, the assessment session was repeated to measure changes.

### Variables

#### Descriptive outcomes

Several descriptive (age, height, weight, and body mass index) and medical and oncological variables (years since diagnosis, months since metastasis diagnosis, affected breast side, type of surgical intervention, metastatic disease site, previous cancer treatment, and current treatment) of the participants were collected.

#### Patient-Reported Outcomes (PRO)

##### European Organization for Research and Treatment of Cancer Breast Cancer-Specific Quality of Life questionnaire (QLQ-BR23)

The quality of life was assessed using the QLQ-BR23 scale, which contains a 4-point scale of 23 items (from 1, not at all, to 4, very much). The final score can be linearly converted to a 100-point scale. This scale has shown good reliability (Cronbach’s α = 0.46–0.94)^33^.

##### Piper Fatigue Scale-Revised (PFS-R)

Cancer-related fatigue is common in patients who have or have had cancer. The PFS-R is a commonly used multidimensional fatigue measure in the cancer research field. This scale includes 22 items with scores from 0 to 10. The revised version of this scale was included in this study, which transfers the total score (0–220) to a scale from 0 to 10 (0 = none, 1–3 = mild, 4–6 = moderate, 7–10 = severe)^34^. The Spanish version used in this study has shown high reliability (Cronbach’s α = 0.96) in people with cancer^35^.

##### Upper Limbs Functional Index (ULFI)

The ULFI index evaluates the upper limb functional capacity and the participation of the subjects^36^. Each participant has to fill 25 items transferable to a 100-point scale (100, best score, and 0, worst score). This study used the Spanish version of the questionnaire, which has high reliability and validity^37^.

##### Lower Limbs Functional Index (LLFI)

The LLFI questionnaire has been designed for assessing the functional capacity of the lower limbs and the social participation^38^. It includes 25 items with different options. The LLFI yields a total score (between 100, best score, and 0, worst score)^38^. The Spanish version of the LLFI index has shown high internal consistency (α = 0.91) and reliability (ICC = 0.96)^39^.

#### US outcomes

The biceps brachii and brachialis (BB) and quadriceps (Q) muscles were analyzed by US, obtaining images of each location and analyzing different parameters. In each of the two locations (BB and Q) an image capture was made in a contraction situation and another in a non-contraction (non-con) situation. Additionally, measurements were developed at baseline (before intervention) and after 12 weeks of exercise intervention (after intervention). The following variables were analyzed:

- Thickness: Thickness refers to the width of the muscle or subcutaneous fat tissue (FT) calculated through the US image’s measurement. To standardize the measurement, it is calculated using a perpendicular line to the horizontal axis on the vertical axis from the midpoint of the bone (femur or humerus). For the muscle, this line was placed between the bone and the superior fascia. For the FT, the line was placed between the fascia and the skin. The value was expressed in cm.
- Echo-intensity: Echo-intensity represents the average result of a histogram of the 8-bit grayscale of the selected range of interest. The resultant histogram analyzed all the pixels of the image from 0, black, to 255, white^40^. This outcome has no unit of measurement.

The combination of these variables (thickness and echo-intensity) in different locations and situations, allowed obtaining six variables in each muscle (BB and Q), analyzed tissue (muscle and FT), and time (before and after intervention): non-con thickness, non-con echo-intensity, contraction thickness, contraction echo-intensity, Difference Thickness and Difference echo-intensity.

The 2D ESAOTE MyLab25Gold (Esaote SpA, Genova, Italia) ultrasound was used to capture images using a 5 cm linear array transducer. A frequency of 12 Hz and a gain of 70% were used. For the evaluation, the patient was placed sitting on an examination table. The evaluator, placed in front of the subject, held the transducer with one hand and the participant's hand or leg with the other one.

**Figure 1:**
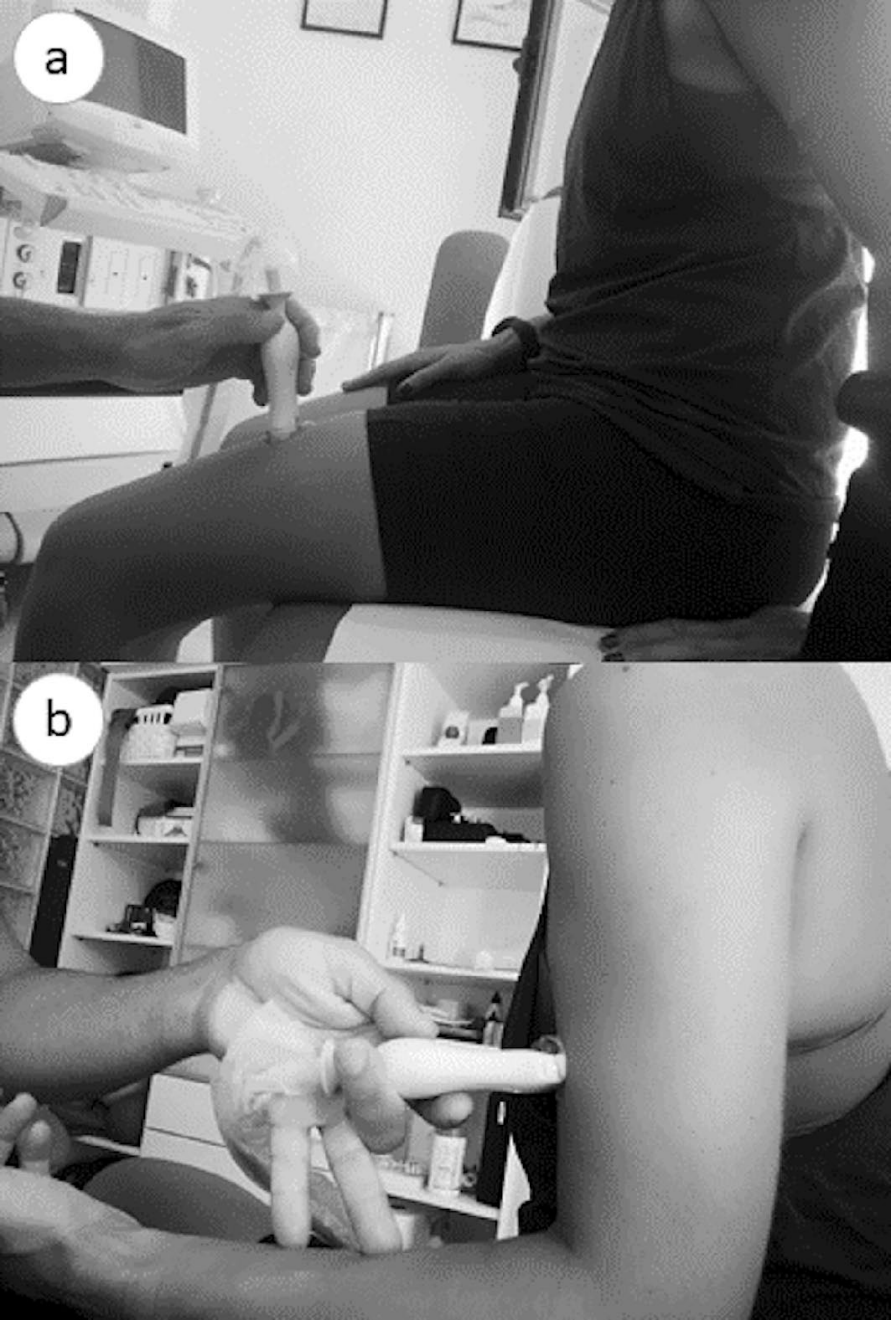
Position of the patient and the ultrasound device for ultrasound assessment of the quadriceps (**a**) and the biceps brachii and brachialis (**b**).

First, thigh images were captured, in non-contraction and contraction. To achieve an adequate capture of a static image in a contraction state, the participant resisted isometrically with a muscular contraction of 5 s. Next, the same process was performed on the arm. At both locations, the transducer was positioned transversely to the direction of the fibers. In the thigh, participants were seated in a chair with their hip and knee at 90° of flexion, and the transducer was placed 15 cm from the upper pole of the patella, in the center of the thigh (Fig. 1a)^25^. In the arm, the evaluator located the main muscle belly of BB, and transducer was placed at the mid-point and anterior part of the humerus^41^. The arm was at rest with the elbow flexed at 90º (Fig. 1b).

All images were exported to a bmp file with a resolution of 800 × 652 px and 96 dpi and were processed and analyzed using MATLAB software (Version R2018b, MathWorks, Natick, USA), as detailed in the section below.

### US data processing and analysis

**Figure 2:**
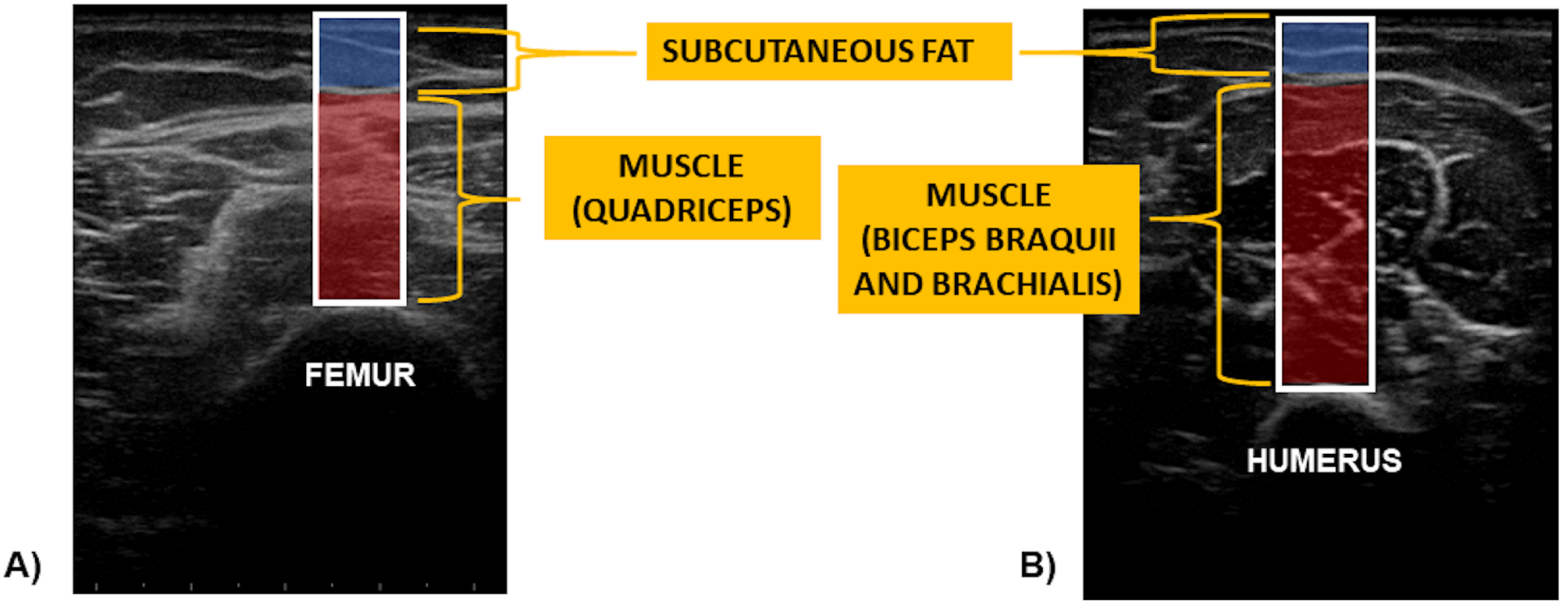
Range of interest (white rectangle) and areas selection of sample images (**A**: quadriceps; **B**: biceps/brachialis).

From the images obtained by ultrasonography, processing and analysis of the same were carried out using an own MATLAB code created specifically for this project. The first function of this code is to allow the researcher to select a range of interest with a width of 1 cm and a height from the cortical bone to the superficial layer of the skin (Fig. 2). Once the range of interest is selected, the code converts the image to grayscale, and the operator manually selects the muscular area (tissue between fascia and bone) and the subcutaneous FT (area between fascia and superficial layer) (Fig. 2). The code analyzes echo-intensity and measures muscle and FT thickness using the selected areas,

### Bias

In an effort to reduce execution and methodological bias, data analysis was conducted by an expert and blinded researcher.

### Statistical methods

The data sets were tabulated and processed using SPSS for Windows (version 25.0, SPSS Inc., Chicago, IL, USA). Quantitative variables were presented using the mean and standard deviation. The qualitative variables were described using absolute and relative frequencies (percentages). In the case of quantitative variables, a bivariate analysis was used to express the mean differences using the T-Student parametric test for dependent samples between before and after intervention outcomes to assess responsiveness. The level of statistical significance established was p > 0.05; determining a 95% confidence interval of the differences in the means of both study groups.

Inferential statistics were carried out between PRO and US outcomes with all before and after intervention measurements, using the Pearson r correlation coefficient. The correlation was considered poor (r < 0.49), moderate (r = 0.50–0.74), or strong (r > 0.75)^42^. A value of p < 0.05 was considered a signal of statistical significance. Additionally, a linear regression analysis was carried out with those PRO with significant correlations with US outcomes in order to found the best regression model.

## Results

### Participants

Thirteen women with MBC were voluntarily included in the study. Participants’ descriptive, medical, and oncological variables are shown in Table 1. All subjects completed the intervention with attendance to at least 70% of the sessions.

**Table 1:** Participant descriptive, medical and oncological variables (n = 13).

	Mean (SD)	Min–max
Age (years)	48.69 (7.20)	40–59
Height (m)	1.63 (0.06)	1.57–1.79
Weight (kg)	74.78 (17.90)	51–119.50
BMI (kg/m^2^)	27.12 (5.76)	19.84–36.72
Years since diagnosis	2.67 (0.81)	1.10–3.00
Months since metastasis diagnosis	26.25 (36.02)	0.50–180.00

	Outcome	Value (%)
Surgery	Mastectomy	7 (53.85%)
	Breast-conserving	2 (15.38%)
	None	4 (30.77%)
Systemic treatment	Chemotherapy	13 (100.00%)
	Radiotherapy	4 (30.77%)
	Hormone therapy	11 (84.62%)
	Monoclonal antibody	6 (46.15%)
Metastatic disease site	Visceral (liver, lung or CNS)	13 (100.00%)
	Non-visceral	10 (76.92%)
	Visceral and Non-visceral	10 (76.92%)
Bone metástasis	Subjects with bone metastastis	10 (76.92%)
	Spine	8 (x%)
	Pelvis	5 (38.46%)
	Thorax	5 (15.38%)
	Humerus	2 (38.46%)
	Femur	5 (38.46%)
Type of bone metastases	Mixed	5 (38.46%)
	Osteoblast	4 (30.77%)
	Osteolytic	1 (7.69%)
Current treatment	Chemotherapy	4 (30.77%)
	Radiotherapy	4 (30.77%)
	Hormone therapy	8 (61.48%)
	Monoclonal antibody	4 (30.77%)

BMI: body mass index; CNS: central nervous system.

### Outcome data and main results

Table 2 includes the results of the intragroup analyzes of the US outcomes and PRO before and after intervention. Regarding PRO, no significant changes were observed in QoL, cancer-related fatigue, and upper and lower limbs function.

**Table 2:** PRO and US imaging results before and after intervention.

	Variables	Before intervention (N = 13)	After intervention (N = 13)	Difference
	QLQ-BR 23	40.72 (11.16)	39.23 (10.62)	− 1.50
	PFS-R	3.98 (2.83)	3.28 (1.82)	− 0.70
	LLFI	69.64 (29.12)	67.87 (31.49)	− 1.76
	ULFI	72.23 (17.31)	72.76 (20.48)	− 0.54
**Quadriceps**				
Muscle	Non-con thickness (cm)	2.48 (0.41)	2.18 (0.33)	0.30*
	Non-con echo-intensity	70.72 (35.11)	72.03 (0.41)	1.31
	Contraction thickness (cm)	2.65 (0.54)	2.59 (0.47)	− 0.05
	Contraction echo-intensity	75.56 (41.01)	89.62 (46.45)	14.06
	Dif thickness (cm)	0.17 (0.38)	0.41 (0.53)	0.24
	Dif echo-intensity	4.84 (11.37)	17.59 (30.73)	12.74
Fat tissue	Non-con thickness (cm)	1.51 (0.57)	1.41 (0.55)	− 0.10
	Non-con echo-intensity	41.10 (20.44)	46.92 (24.23)	5.81
	Contraction thickness (cm)	1.27 (0.45)	1.16 (0.43)	− 0.10
	Contraction echo-intensity	34.48 (16.86)	38.54 (20.45)	4.06
	Dif thickness (cm)	− 0.23 (0.21)	− 0.24 (0.27)	− 0.01
	Dif echo-intensity	− 6.62 (6.33)	− 8.37 (9.47)	− 1.75
**Biceps brachii and brachialis**				
Muscle	Non-con thickness (cm)	2.60 (0.65)	2.81 (0.64)	0.21
	Non-con echo-intensity	72.88 (33.02)	86.19 (38.13)	13.31
	Contraction thickness (cm)	2.97 (0.55)	2.90 (0.51)	− 0.07
	Contraction echo-intensity	89.07 (48.58)	86.15 (35.42)	− 2.92
	Dif thickness (cm)	0.36 (0.67)	0.09 (0.45)	− 0.28
	Dif echo-intensity	16.19 (22.69)	− 0.04 (17.03)	16.22*
Fat tissue	Non-con thickness (cm)	1.00 (0.55)	0.82 (0.30)	− 0.18
	Non-con echo-intensity	32.51 (28.21)	25.91 (15.07)	− 6.60
	Contraction thickness (cm)	0.59 (0.29)	0.79 (0.51)	0.18
	Contraction echo-intensity	18.68 (14.74)	24.14 (20.09)	5.46
	Dif thickness (cm)	− 0.40 (0.35)	− 0.03 (0.38)	0.38**
	Dif echo-intensity	− 13.83 (15.64)	− 1.77 (14.40)	12.1

Dif, Difference; QLQ-BR, Quality of Life Questionnaire Breast Cancer; PFS-R, Piper Fatigue Scale-Revised; LLFI, Lower Limb Functional Index; Non-con, no contraction; ULFI, Upper Limb Functional Index; PRO: Patient-Reported Outcomes; US: Ultrasound imaging. *p < 0.05; **p < 0.01.

In the Q muscle, a decrease in thickness occurred during non-con (0.30) and contraction (0.05) situation, although this decrease was only significant in non-con. The rest of the muscle thickness variables decreased but represented a small and non-significant difference. There were no significant findings in Q FT outcomes (see Table 2).

In BB muscle, a significant decrease was found between the outcomes difference echo-intensity before and after the intervention (16.22, p = 0.03). This outcome describes the difference between non-con and contraction, so this difference decreased in the measurement at 3 months with respect to the initial measurement. Significant findings were also found in thickness difference (0.38 cm; p = 0.007).

Among the four PROs analyzed, only the QLQ-BR23 questionnaire showed significant correlations with US outcomes. The significant correlations between the results obtained in this PRO and the US outcomes from all measurements are presented in Table 3. The best correlations were found between the QLQ-BR 23 questionnaire and Q Non-con echo-intensity (r = − 0.41, p < 0.05), Q FT Non-con Thickness (r = 0.44, p < 0.05), BB Muscle Non-con Thickness (r = 0.55, p < 0.01), BB Muscle Contraction Thickness (r = 0.61, p < 0.01) (Table 3).

**Table 3:** Significant correlations (r) between the QLQ-BR 23 questionnaire (PRO) and US outcomes.

US outcomes	
Q muscle non-con echo-intensity	− 0.41*
Q FT non-con thickness	0.44*
BB muscle non-con thickness	0.55**
BB muscle contraction thickness	0.61**

BB, biceps brachii and brachialis; QLQ-BR 23, Quality of Life Questionnaire Breast Cancer; Q, quadriceps; EI, echointensity; PRO: Patient-Reported Outcomes; non-con, non-contraction; US, Ultrasound imaging.

*p < 0.05; **p < 0.01.

The best model found in the linear regression analysis from those outcomes with significant correlations is presented in Table 4. Multiple linear regression analysis showed that BB Muscle Non-con Thickness after adjusting by age and weight, explained 70% of QLQ-BR 23 variance.

**Table 4:** Multiple regression analysis about the Quality of Life Questionnaire Breast Cancer (QLQ-BR 23).

Dependent variables	Predictor variables	Standardized β	R	R^2^
QLQ-BR 23	Biceps brachii and brachialis muscle non-contraction thickness	0.49*	0.84	0.70*
	Age	0.22		
	Weight	0.64*		

QLQ-BR, Quality of Life Questionnaire Breast Cancer.

*p < 0.05.

## Discussion

The present study analyzed the responsiveness of muscle thickness and echo-intensity extracted from the US images as new biomarkers and their relationship with functional and PRO in MBC patients. As authors are aware, this is the first study to analyze these outcomes after an exercise intervention in this oncology population. As the main finding, significant differences were found in non-contraction muscle Q thickness and echo-intensity Difference in BB muscle before and after the intervention. However, changes in FT and PRO did not reach statistical significance (Table 2). That is to say, in MBC patients, proposed US biomarkers related to muscle architecture showed greater responsiveness than those related to FT and PROs. At the light of these results, MBC could be assessed by a non-invasive and low-cost tool such as US to measure the effect of exercise, which is one of the most recommended interventions in the oncology field^43–46^. In addition, these findings reinforce the analysis of muscle architecture by US in a population not previously assessed with these variables.

A combination of functional tests and PRO are used to describe function in MBC patients^47^, and they are usually employed to measure the effect of exercise intervention in patients with advanced cancer^48^ and those suffering from metastasis^49–51^. Changes in body composition in the cancer population are mainly assessed by plethysmography, dual-energy X-ray absorptiometry (DXA), BMI, or skinfold thickness^48^. Regarding US outcomes, some like the physiological cross-sectional area has been used to measure adaptations after exercise interventions in older adults^22^. Biomarkers proposed in the present study, namely muscle thickness and echo-intensity, are useful to measure sarcopenia in elders^25^. However, given the novelty of the presented outcomes in this oncology, comparison with previous research is not possible. Proposed US biomarkers from the present study may add information about muscle and FT to traditionally and widely employed methods.

It should be noteworthy that MBC patients did not present improvements in Q muscle thickness, which could be influenced by several factors. On the one hand, previous literature has shown that exercise interventions preserve physical function in prostate cancer patients with bone metastases. Compared with the control group, patients showed function improvements measured by PRO and functional tests after 3 months^32^. Specifically, 5 out of our 13 patients had bone metastasis in the femur. Besides, 3 months of exercise interventions may ameliorate deterioration, but further time could be required to observe differences before and after exercise. In this regard, an increase in strength during the first 3 months of training are mainly due to neural adaptations^52^. A systematic review analyzing the chronic effect of strength exercises in BC survivors reported that only 1 out of 10 studies showed a significant change in lean mass^53^. In addition, this study measured the effects 6 and 12 months post-intervention^54^ compared to the 3 months evaluated in our study. On the other hand, to guarantee safety, exercise adaptations may have limited possible changes in muscle architecture, as a high load is required for a hypertrophic response^55^. In this regard, literature concerning exercise intervention in patients with bone metastases is quite limited to exercise feasibility, avoiding affected areas^32^ or at low intensities^50^ given the risk of fracture^56^. Another factor that may have influence changes in muscle mass is cancer treatment and cancer itself, as they induce muscular adaptations that counteract those induced by exercise training^57^. As shown in Table 1, all patients from the present study were treated with chemotherapy, and four were under chemo treatment during the intervention. Otherwise, previous literature shows that, in patients undergoing cancer treatment, resistance exercise interventions reduce body fat but only maintain muscle mass despite strength gaining^58^. Those findings concur with results from the present study, in which fat tissue from lower and upper limbs tend to decrease.

In contrast to Q, patients tended to increase non-con muscle thickness and echo-intensity in BB. BC patients who had undergone breast surgical intervention believe that lifting heavy objects would increase the chance of developing breast cancer-related lymphedema^59^ and leading to fear and avoidance of the affected arm^60,61^. MBC patients tend to have lower function levels, represented as lower grip strength and a restricted shoulder range of motion^47^. In the present sample, 7 and 2 out of 13 patients had had a mastectomy and conserving surgery, respectively. As a result, exercise may have been intense enough to keep function in the upper limbs (similar ULFI punctuation and a slight increase in BB non-con thickness), but not in the lower limbs (− 1.76% LLFI punctuation) in the present sample. Present results concur with a meta-analysis that found exercise in patients with cancer interventions have a large effect on upper extremities than lower limbs, as upper limbs may be more susceptible to a decrease in muscle strength due to physical inactivity during treatment, while activity of daily living such as walking may attenuate the decrease in the lower limbs^62^.

It should be highlighted that QLQBR-23 significantly correlated with most US outcomes (Table 3). This finding supports the importance of US outcomes, as health-related QoL predicts mortality in the BC population^63^. Besides, muscle mass has been associated with mortality^64^. Specifically, the strongest association was found in those measured in the upper limb (0.55** and 0.61** for BB non-con and contraction thickness, respectively). This concurs with results from the linear regression (Table 4), in which this US outcome explained 70% of QLQ-BR 23 variance once corrected by age and weight.

Recent research proposes muscle mass preservation as a strategy to reduce treatment-related toxicities^65^ and manage cancer cachexia, which is associated with mortality^66^. In this regard, exercise intervention is currently proposed as a treatment aimed at counteracting muscle wasting and deterioration^57,58,67^. There is evidence enough to prescribe exercise-dose depending on the symptom or secondary effect aimed at improving during cancer survivorship^68^. However, dose targeting muscle mass preservation or hypertrophy have not been elucidated among cancer survivors yet^53^. As previously mentioned, in MBC, muscle changes may be influenced by several factors such as treatment or exercise adaptation due to bone metastasis. As a consequence, the exercise-dose from the present intervention may not be intense enough to produce changes. On the other hand, given the relationship between hormonal status and outcomes such as BMI, FT inflammation and systemic markers^69^, future research should study the relationship of presented US biomarkers and systemic markers related to inflammation status in women with different hormonal treatment and after exercise intervention^70^. In addition, future research should include interventions with a longer duration to study if further time is required to see statistical changes in presented outcomes. Lately, future randomized controlled trials should study differences between the presented US biomarkers with control groups.

## Conclusion

The present study analyzed US biomarkers' responsiveness to an exercise intervention in MBC patients and its relationship with functional and PRO. US biomarkers related to muscle architecture (muscle thickness) showed greater responsiveness than those related to FT and PROs. After the intervention, improvements were observed in upper limb US outcomes, which mostly correlated with QLQ-BR 23 and explained 70% of its variance once corrected by age and weight. Future research should address proposed US biomarkers to measure the effect of exercise interventions in MBC patients.

## Data Availability

The datasets used and/or analysed during the current study are available from the corresponding author upon reasonable request.

## Abbreviations

BB: Biceps brachii and brachialis
BC: Breast cancer
BMI: Body mass index
DALYs: Disability-adjusted life-years
DXA: Dual energy X-ray absorptiometry
FT: Fat tissue
MBC: Metastatic breast cancer
Non-con: Non-contraction
LLFI: Lower Limbs Functional Index
PFS-R: Piper Fatigue Scale-Revised
PRO: Patient-reported outcomes
Q: Quadriceps
QLQ-BR 23: Quality of Life Questionnaire Breast Cancer
QoL: Quality of life
ULFI: Upper Limbs Functional Index
US: Ultrasound imaging
